# Macrolide-Lincosamide Resistance and Virulence Genes in *Staphylococcus aureus *Isolated from Clinical Specimens in Ardabil, Iran

**DOI:** 10.30699/IJP.2023.1987077.3049

**Published:** 2023-10-15

**Authors:** Meysam Manouchehrifar, Farzad Khademi, Hadi Peeri Doghaheh, Shahram Habibzadeh, Mohsen Arzanlou

**Affiliations:** 1 *Department of Microbiology, School of Medicine, Ardabil University of Medical Sciences, Ardabil, Iran*; 2 *Department of Infectious Diseases, School of Medicine, Ardabil University of Medical Sciences, Ardabil, Iran*

**Keywords:** iMLSB resistance Lincosamide, Macrolide, Spa typing, Staphylococcus aureus, Virulence genes

## Abstract

**Background & Objective:**

*Staphylococcus aureus* causes various hospital- and community-acquired infections. This study aimed to investigate the phenotypic and genotypic characteristics of erythromycin and inducible clindamycin resistance, virulence gene profiles, and *spa* types of *S. aureus* isolates collected from patients in Ardabil Province, Iran.

**Methods:**

A total of 118 clinical *S. aureus* isolates, including 50 (42.4%) methicillin-resistant *S. aureus* (MRSA) and 68 (57.6%) methicillin-susceptible *S. aureus* (MSSA) strains, were investigated. Resistance patterns were determined by the disk diffusion method and minimum inhibitory concentration (MIC) test. Inducible macrolide-lincosamide-streptogramin B (iMLSB) resistance was detected using D-test method. The polymerase chain reaction (PCR) was used to identify the virulence and resistance-encoding genes. Additionally, the *spa* types of the isolates were determined using the PCR, followed by sequencing.

**Results:**

In total, 49.1% (58/118) and 44% (52/118) of the isolates were resistant to erythromycin and clindamycin, respectively. Overall, 13.5% (16/118) of the isolates showed the iMLSB resistance phenotype. The *ermC* gene (72.4% [42]) was the most frequent erythromycin resistance-encoding gene, followed by *ermA* (60.3% [35]), *ermB *(60.3% [35]), *ermTR* (51.7% [30]), and *msrA* (15.5% [9]) genes among erythromycin-resistant isolates. The virulence genes *hla*, *hld*, *sea*, *LukS PV*, *tst*,* seb, sed*, *eta*, *sec*, and *etb *were detected in 93.2%, 74.5%, 70.3%, 32.2%, 29.6%, 17%, 8.5%, 8.5%, 5.9%, and 4.2% of the isolates, respectively. Ten different *spa* types were identified for 58 erythromycin-resistant *S. aureus* strains, of which t030 and t078 types were the most common types.

**Conclusion:**

A high frequency of macrolide- and lincosamide-resistant *S. aureus* isolates with different genetic backgrounds of resistance and virulence may be found in patients in Ardabil Province, Iran.

## Introduction

*Staphylococcus aureus* is one of the most common microorganisms found in the skin and mucous membranes of healthy children and adults. However, it is associated with several hospital- and community-acquired infections, including toxigenic diseases (such as staphylococcal scalded skin syndrome, food poisoning, and toxic shock syndrome) and suppurative infections (such as impetigo, folliculitis, furuncles, and carbuncles) (1). *S. aureus* pathogenicity depends on the multiple virulence determinants, as well as their ability to acquire resistance to various antibiotics (2).

Several important toxins, including staphylococcal enterotoxins (SEs), exfoliative toxins (ETs), Panton–Valentine leukocidin (PVL), hemolysins, and toxic shock syndrome toxin-1 (TSST-1), are excreted by *S. aureus*, contribute to the pathogenesis of staphylococcal diseases (3).

The increasing incidence of methicillin-resistant *S. aureus* (MRSA) strains is now an important health challenge worldwide. Most MRSA isolates show resistance to β-lactam antibiotics and several other classes of antibiotics (4). Macrolide-lincosamide-streptogramin B (MLSB) antibiotics are a major alternative to β-lactam antibiotics for the treatment of infections caused by gram-positive bacteria. Clindamycin is a bacteriostatic antibiotic suitable for the treatment of staphylococcal infections (especially skin and soft tissue infections) because it penetrates well into most tissues (5). However, the extensive use of MLSB antibiotics has led to the emergence and spread of resistant bacterial species worldwide (6). MLSB antibiotics differ structurally but exhibit similar functions against bacteria through inhibition of the protein synthesis via binding to ribosomal 23s rRNA (5). In total, 3 different resistance mechanisms are involved in resistance to MLSB antibiotics in *S. aureus* strains, including enzymatic alteration of the target site of MLSB drugs on ribosomes (encoded by *erm* genes), extruding antibiotics out of the bacteria (efflux pumps; encoded by the *msrA* gene), and enzymatic modification of lincosamides (encoded by the *inuA* gene) (7). Among them, the most common resistance mechanism is associated with *erm* genes, producing a methylase enzyme to methylate ribosomal 23s rRNA (8). According to the* erm* gene-associated resistance mechanism, *S. aureus* shows 3 different resistance phenotypes: (i) the constitutive MLSB (cMLSB) phenotype (isolates are resistant to both erythromycin and clindamycin), (ii) inducible MLSB (iMLSB) phenotype (isolates become resistant to clindamycin in the presence of an inducing substance like erythromycin), and (iii) MS phenotype (isolates are resistant to erythromycin but sensitive to clindamycin) (9). Among these resistance phenotypes, inducible clindamycin resistance (iMLSB) is a significant clinical issue as it is not detectable by conventional methods, and inaccurate identification may lead to treatment failure (10). Therefore, for the identification of inducible clindamycin resistance, a combination of reliable phenotypic and genotypic methods is required (11).

In Ardabil Province, little is known about the prevalence of resistance to MLSB antibiotics and the virulence properties of *S. aureus* isolates. This study was performed for the first time to determine (i) the frequency of resistance to erythromycin and clindamycin, (ii) occurrence of inducible clindamycin resistance, (iii) distribution of virulence genes, and (iv) clonal relationship of isolates using the *spa* typing method among *S. aureus* strains isolated from patients.

## Material and Methods

**Bacterial Isolates**

A total of 118 *S. aureus* isolates, including 50 MRSA and 68 MSSA strains, were used in this cross-sectional study. The strains were obtained from patients admitted to 4 teaching hospitals in Ardabil Province between February 2017 and June 2018. The strains were stored frozen at -70 °C in Trypticase soy broth (Merck, Germany) along with 15% glycerol (Kimia, Iran) until tested.

**Antimicrobial Susceptibility Testing**

The minimum inhibitory concentrations (MICs) of erythromycin (Bio Basic, Ontario, Canada) were determined using the agar dilution method (12). The isolates with an MIC of ≥8 μg/μL were considered erythromycin-resistant (13). The disk diffusion method was used to evaluate the susceptibility of isolates to clindamycin using a 2-µg clindamycin disk. The D-test method was used to determine inducible clindamycin resistance (iMLSB) in erythromycin-resistant and clindamycin-sensitive isolates. This was performed using erythromycin (15 µg) and clindamycin (2 µg) disks (Padtan Teb Company, Tehran, Iran). All tests were performed and interpreted in accordance with the Clinical and Laboratory Standards Institute (CLSI) guidelines (13).

**Detection of** **Resistance and Virulence** **Genes**

Total DNA was extracted from *S. aureus* isolates using a commercial kit (CinnaGen, Tehran, Iran) according to the manufacturer’s instructions. The extracted total DNA was evaluated and quantified using a NanoDrop spectrophotometer (Thermo Scientific, USA) and then stored frozen at -20°C until use. Using the polymerase chain reaction (PCR),* S. aureus *isolates were further confirmed by amplification of the *nuc* gene (encoding a thermostable nuclease enzyme) using specific primers (Table 1). The PCR for the amplification of the *nuc* gene was performed in a 25-μL PCR PreMix (CinnaGen, Tehran, Iran) with 10 pmol of each primer under the following conditions: 1 cycle of initial denaturation at 95°C for 5 min, 34 cycles of denaturation at 95°C for 30 s, annealing at 58°C for 30 s, extension at 72°C for 45 s, and 1 cycle of final elongation at 72°C for 7 min. Similarly, singleplex PCRs were performed for the identification of *msrA *and *erm* (*erm A*, *erm B, erm C, *and *erm TR) *genes, as well as virulence genes *sea*, *seb*,* sec*,* sed*,* eta*,* etb*,* LuX/F-PV*,* hla*,* hld,* and *tst*, in 25-μL final volumes as mentioned earlier with different annealing temperatures (Table 1). To confirm the identity of the amplicons, the nucleotide sequence of a randomly selected PCR product from resistance and virulence genes was determined (Bioneer, Daejeon, South Korea). Sequences were aligned and analyzed using the BLAST program available at the National Center for Biotechnology Information (NCBI). PCR products were analyzed by electrophoresis on a 1.5% agarose gel (SinaClon, Tehran, Iran) and stained with DNA-safe stain (SinaClon, Tehran, Iran). Bands were visualized under UV light (Uvitec, Cambridge, UK).

***Spa*** **Typing**

The *spa* types of randomly selected erythromycin-resistant *S. aureus* strains were determined using the PCR-sequencing method with specific primers (Table 1). The PCR conditions were similar to the amplification of other genes described earlier in the text with a 62°C annealing temperature. The sequences of one strand of the amplicons were determined at Bioneer Company. The *spa* gene sequences were analyzed by the online software (http://www.spaserver.ridom.de); accordingly, the isolates were assigned to particular spa types. 

**Statistical Analysis**

The Chi-square test was used to measure the statistical association between resistance characteristics and isolate types (MRSA/MSSA). P-values less than 0.05 were considered statistically significant. 

**Table 1:** The primers sequences used in this study

References	Annealing temperatures (°C)	Product size (bp)	Primer sequence (5 → 3)	Gene
	60	372	F: TCAGGAAAAGGACATTTTACC R: ATATAGTGGTGGTACTTTTTTGAGC	***ermA***
	60	494	F: GGTAAAGGGCATTTAACGAC R:GGTAAAGGGCATTTAACGAC	***ermB***
	61	184	F: CTTGTTGATCACGATAATTTCC R: TAGCAAACCCGTATTCCACG	***ermC***
	61	375	F: TCAGGAAAAGGACATTTTACC R: AAAATATGCTCGTGGCAC	***ermTR***
	57	164	F: TCCAATCATTGCACAAAATC R: CAATTCCCTCTATTTGGTGGT	***msrA***
	57	209	F: CTGATTACTATCCAAGAAATTCGATTG R: CTTTCCAGCCTACTTTTTTATCAGT	***hla***
	59	111	F: AAGAATTTTTATCTTAATTAAGGAAGGAGTG R: TTAGTGAATTTGTTCACTGTGTCGA	***hld***
	59	102	F: GCAGGGAACAGCTTTAGGC R: GTTCTGTAGAAGTATGAAACACG	***sea***
	59	164	F: ACATGTAATTTTGATATTCGCACTG R: TGCAGGCATCATGTCATACCA	***seb***
	58	278	F: GTGGTGAAATAGATAGGACTGC R: ATATGAAGGTGCTCTGTGG	***sed***
	58	284	F: CTTGTATGTATGGAGGAATAACAA R: TGCAGGCATCATATCATACCA	***sec***
	59	443	F: ATCATTAGGTAAAATGTCTGGACATGATCC R: GCATCAASTGTATTGGATAGCAAAAGC	***luk S***
	58	326	F: GCTTGCGACAACTGCTACAG R: TGGATCCGTCATTCATTGTTAT	***tst***
	58	93	F: GCAGGTGTTGATTTAGCATT R: AGATGTCCCTATTTTTGCTG	***eta***
	58	226	F: ACAAGCAAAAGAATACAGCG R: GTTTTTGGCTGCTTCTCTTG	***etb***
	58	279	F: GCGATTGATTGATGGTGATACGGTT R AGCCAAGCCTTGACGAACTAAAGC:	***nuc***
	61	Variable	F: AAAATCGATGGTAAAGGTTGGC R: AGTTCTGCAGTACCGGATTTGC	***spa***
	55	Variable	F: ATGTAAGCTCCTGGGGAATTCAC R: AAGTAAGTGACTGGGGTGAGCG	**ERIC**

**Table 2:** Frequency of MLSB resistance phenotypes among strains of *S. aureus*

Phenotype	MRSA, N = 50 n (%)	MSSA, N = 68 n (%)	Total N = 118 n (%)
ER-R, CL-R (cMLS_B_)	25 (50) *	2 (2.9)	27 (22.9)
ER-R, CL-S (iMLS_B_)	5 (10)	11 (16.2) *	16 (13.5)
ER-R, CL-S (MS) ER-S, CL-R	8 (16) 3(6)	7 (10.3) 6(8.8)	15 (12.7) 9 (7.6)

Abbreviations: MRSA; methicillin-resistant* S. aureus*, MSSA;methicillin-susceptible* S. aureus*, ER-R; Erythromycin resistant , ER-S; Erythromycin susceptible, CL-R; Clindamycin resistant, CL S; Clindamycin susceptible. * P-value ≤ 0.05 was considered statistically significant

## Results

According to the disk diffusion assay, 49.1% (58/118) and 44% (52/118) of the *S. aureus* isolates were resistant to erythromycin and clindamycin, respectively. Furthermore, the MICs of erythromycin ranged from 0.25 to 512 µg/mL. Consistent with the disk diffusion assay results, 58 isolates showed MICs over the resistance breakpoint (≥8 µg/mL).

According to the D-test results, 27/118 (22.9%) isolates showed the cMLSB resistance phenotype, 16/118 (13.5%) isolates showed the iMLSB phenotype, and 15/118 (12.7%) isolates showed the MS phenotype (Table 2). The rate of the cMLSB resistance phenotype was significantly higher in MRSA strains (*P*≤0.05), while the iMLSB phenotype was higher in MSSA strains (*P*≤0.05).

**Fig. 1:**
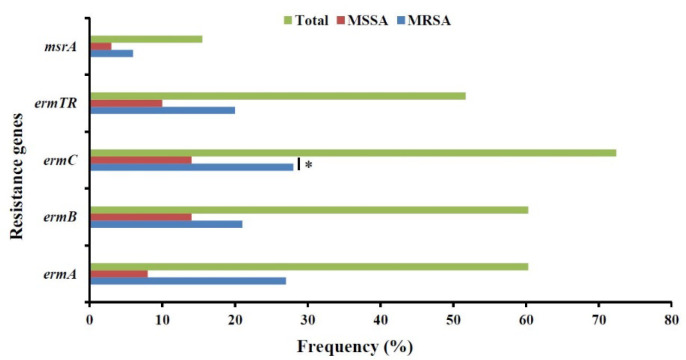
Distribution of macrolide resistance encoding genes in erythromycin-resistant strains of *S. aureus* collected from clinical specimens in the teaching hospitals in Ardabil, Northwest, Iran

Erythromycin resistance genes were detected using PCR in 58 erythromycin-resistant isolates. Thirty-five (60.3%), 35 (60.3%), 42 (72.4%), 30 (51.7%), and 9 (15.5%) of the erythromycin-resistant strains showed the presence of *ermA*, *ermB*, *ermC*, *ermTR*, and *msrA* genes, respectively (Figure 1). The occurrence of *ermA* and *ermC* genes was significantly higher in MRSA isolates. Nineteen different profiles of macrolide-resistance encoding genes were observed in the *S. aureus* isolates. Profiles RP7, RP12, and RP18 contained the highest percentage of macrolide antibiotic resistance genes, while in 5 macrolide-resistant isolates (8.6%), all resistance genes were observed (RP19; Table 3).

**Detection of Virulence Genes**

**Fig. 2:**
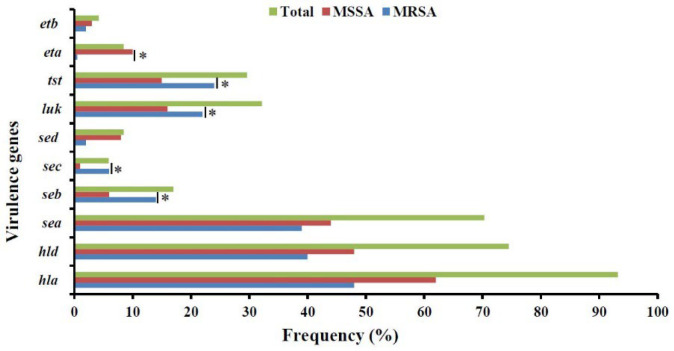
Frequency distribution of virulence encoding genes among MRSA and MSSA strains collected from clinical specimens in the teaching hospitals in Ardabil, Northwest, Iran

Abbreviations: MRSA-methicillin-resistant* S. aureus*, MSSA-methicillin-susceptible* S. aureus*.

Figure 2 shows the distribution of virulence-encoding genes in MRSA and MSSA isolates. In the present study, hemolysin-encoding genes *hla* and *hld* were detected in 110 (93.2%) and 88 (74.5%) isolates, respectively. Enterotoxin-encoding genes *sea*, *sec,* *seb, *and* sed* were observed in 83 (70.3%), 7 (5.9%), 20 (17%), and 10 (8.5%) isolates, respectively. Exfoliative toxin-encoding genes *eta* and *etb* were detected in 10 (8.5%) and 5 (4.2%) isolates. PVL- and TSST-1–encoding genes (*lukSF-PV* and *tst*) were found in 38 (33.2%) and 35 (29.6%) isolates, respectively. In comparison, the incidence of *seb* and *tst* genes was significantly higher in MRSA strains (*P*≤0.05), while the *eta* gene was significantly higher in MSSA isolates (*P*≤0.05). However, a total of 29 different virulence gene profiles were detected in the *S. aureus* isolates, with VP10 as the predominant profile containing *hla, hld, sea,* and *tst* genes (Table 4).

***Spa*** **Typing**

**Fig. 3:**
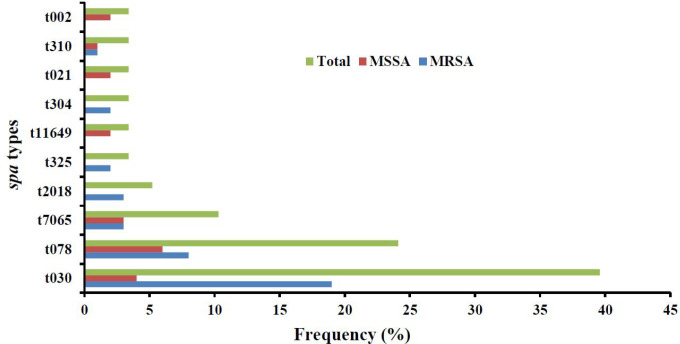
Frequency of *spa *types among MRSA and MSSA strains isolated from patients in the teaching hospitals in Ardabil, Northwest, Iran

Abbreviations: MRSA-methicillin-resistant* S. aureus*, MSSA-methicillin-susceptible* S. aureus*.

Out of the 58 isolates evaluated, a total of 10 *spa* types were identified. The most frequent types were t030 (in 23 isolates [39.6%]) and t078 (in 14 isolates [21.4%]), followed by t7065 (in 6 isolates [10.34%]), t2018 (in 3 isolates [2.17%]), t325 (in 2 isolates [3.44%]), t11649 (in 2 isolates [3.44%]), t304 (in 2 isolates [3.44%]), t021 (in 2 isolates [3.44%]), t310 (in 2 isolates [3.44%]), and t002 (in 2 isolates [3.44%]; see Figure 3).

* P-value of 0.05 or below was considered statistically significant

* P-value of 0.05 or below was considered statistically significant.

**Table 3:** Combination pattern of resistance encoding genes among erythromycin-resistant strains of *S. aureus*

Resistance Profile	Gene (s)	*S. aureus *strains		
		MRSA N = 38, n (%)	MSSA N = 20, n (%)	Total N = 58, n (%)
RP1	*ermA*	1 (2.6)	-	1 (1.7)
RP2	*ermB*	2 (5.3)	-	2 (3.5)
RP3	*ermC*	2 (5.3)	3 (15)	5 (8.6)
RP4	*ermTR*	2 (5.3)	1 (5)	3 (5.2)
RP5	*msrA*	1 (2.6)	-	1 (2.6)
RP6	*ermA+ermB*	1 (2.6)	1 (5)	2 (3.5)
RP7	*ermA+ermC*	6 (15.8)	-	6 (10.3)
RP8	*ermC+ ermTR*	2 (5.3)	1 (5)	3 (5.2)
RP9	*ermB+ermC*	-	2 (10)	2 (3.5)
RP10	*ermB+ msrA*	-	1 (5)	1 (1.7)
RP11	*ermA+ ermTR*	1 (2.6)	-	1 (1.7)
RP12	*ermA+ermB+ermC*	5 (13.1)	2 (10)	7 (12.1)
RP13	*ermA+ermC+ ermTR*	2 (5.3)	1 (5)	3(5.2)
RP14	*ermA+ermB+ ermTR*	2 (5.3)	2 (10)	4 (6.9)
RP15	*ermB+ermC+ msrA*	-	1 (5)	1 (1.7)
RP16	*ermB+ermC+ermTR*	2 (5.3)	2 (10)	4 (6.9)
RP17	*ermB+ ermTR+ msrA*	-	1 (5)	1 (1.7)
RP18	*ermA+ermB+ermC+ermTR*	4 (10.5)	2 (10)	6 (10.3)
RP19	*ermA+ermB+ermC+ermTr+msrA*	5 (13.1)	-	5 (8.6)

Abbreviations: MRSA-methicillin-resistant* S. aureus*, MSSA-methicillin-susceptible* S. aureus*.

**Table 4:** Distribution of toxins gene profiles among erythromycin-resistant strains of *S. aureus*

Virulence Profile	Gene (s)	*S. aureus* strains		
		MRSA N=38, n (%)	MSSA N=20, n (%)	Total N=58, n (%)
VP1	*sea*	1 (2.6)	-	1 (1.7)
VP2	*hla + sea*	2 (5.3)	-	2 (3.5)
VP3	*hla + luk*	1 (2.6)	-	1 (1.7)
VP4	*hld+ sea*	-	1 (5)	1 (1.7)
VP5	*hla +hld + sea*	6 (15.8)	1 (5)	7 (12.1)
VP6	*hla +hld +sed*	-	1 (5)	1 (1.7)
VP7	*hla + sea +luk*	-	1 (5)	1 (1.7)
VP8	*hla + sed +tst*	1 (2.6)	-	1 (1.7)
VP9	*hla +hld + sea + luk*	-	3(15)	3 (5.2)
VP10	*hla +hld + sea + tst*	5 (13.2)	2 (10)	7 (12.1)
VP10	*sec+ hla+hld +sea*	6 (15.8)	-	6 (10.4)
VP11	*hla +hld + sea + seb*	1 (2.6)	1 (5)	2 (3.5)
VP12	*hla +hld + sea + etb*	1 (2.6)	1 (5)	2(3.5)
VP13	*hla +hld + sea + eta*	-	1 (5)	1 (1.7)
VP14	*hla + sea +seb +tst*	1 (2.6)	-	1 (1.7)
VP15	*hla + luk + seb +tst*	2 (5.3)	-	2 (3.5)
VP16	*hla +hld + luk + tst*	2 (5.3)	-	2 (3.5)
VP17	*hla +hld + sea + sed*	1 (2.6)	-	1 (1.7)
VP18	*hla + sea +luk + seb*	1 (2.6)	-	1 (1.7)
VP19	*hla+sea+luk+seb+tst*	-	1 (5)	1 (1.7)
VP20	*sec + hla + sea + luk*	1 (2.6)	-	1 (1.7)
VP21	*hla +hld + sea + eta + tst*	-	1 (5)	1(1.7)
VP22	*hla +hld + sea + luk + tst*	2 (5.3)	1 (5)	3 (5.2)
VP23	*hla +hld + sea + seb + tst*	2 (5.3)	1 (5)	3 (5.2)
VP24	*hla +hld + sea + etb+ seb*	-	1 (5)	1 (1.7)
VP25	*sec+ hla+hld +sea+ luk*	1 (2.6)	-	1 (1.7)
VP26	*sec+ hla+hld +sea+ luk+ seb*	-	1 (5)	1 (1.7)
VP27	*sec+ hla+hld +sea+seb+sed*	1 (2.6)	-	1 (1.7)
VP28	*hla +hld + sea + luk+eta+seb+tst*	-	1 (5)	1 (1.7)
VP29	*hla +hld + sea + luk+seb+sed+ tst*	-	1 (5)	1 (1.7)

## Discussion

Drug resistance and virulence factors are 2 important aspects of *S. aureus* pathogenicity (14). Frequent increases in infections caused by *Staphylococcus* strains and the changes in antibiotic resistance patterns have led to the reuse of effective agents (such as macrolide-lincosamide antibiotics) in the treatment of systemic and localized infections caused by this organism. These drugs, although having different structures, have the same function against *S. aureus* (14). The frequency of erythromycin-resistant *S. aureus* was 49.1% in our study. The rate is slightly higher (40%-43.8%) than results reported from other cities in Iran and lower than the reports from countries such as India (51.7%), Korea (77.5%), and Turkey (60.4%) (15, 16).

Clindamycin is associated with a high absorption capacity; therefore, it is used in the treatment of staphylococcal bone and skin infections (17). Due to the increased resistance to clindamycin among *S. aureus* isolates, the possibility of treatment failure with this antibiotic is not unlikely (18). The rate of clindamycin-resistant *S. aureus* was 44% (in 52 isolates) in Ardabil Province. Findings reported from other cities in Iran and other countries were in a similar range: Tabriz (38%), Tehran (35.6%), Shiraz (36%), and India (34.4%) (19). The emergence of drug-resistant *S. aureus* isolates, including methicillin- and MLSB-resistant strains, is attributed to a combination of factors, including the overuse or misuse of antibiotics and the ability to exchange genetic material through resistance plasmids (20). Because of a similar mechanism of action, cross-resistance in MLSB antibiotics is common in gram-positive cocci (21). Inducible clindamycin resistance may occur in erythromycin-resistant staphylococci due to *erm* genes, which is not detectable through the common disk diffusion method (22). Inducible clindamycin resistance in *S. aureus* strains (iMLSB) was reported between 7%-94% worldwide (23). In this study, the frequency of cMLSB, iMLSB, and MS phenotypes in *S. aureus* isolates was 22.9% (in 27 isolates), 13.5% (in 16 isolates), and 12.7% (in 15 isolates), respectively. In studies conducted in different cities of Iran, the prevalence of cMLSB, iMLSB, and MS phenotypes in *S. aureus* isolates was as follows: in Ghazvin, it was 37% for cMLSB, 6.5% for iMLSB, and 1.3% for MS phenotypes; in Arak, it was 9.5% for cMLSB, 2.5% for iMLSB, and 2.5% for MS phenotypes; in Tehran, it was 88% for cMLSB, 7% for iMLSB, and 3% for MS phenotypes; in Isfahan, it was 32% for cMLSB, 6% for iMLSB, and 6% for MS phenotypes; and in Kerman, it was 51.2% for cMLSB, 8.6% for iMLSB, and 9.3% for MS phenotypes (24-25). The frequency of the iMLSB resistance phenotype in this study was higher than in other studies in Iran, indicating an increased rate of erythromycin antibiotic use in Ardabil. Additionally, in Denmark, Turkey, and Greece, the frequency of the iMLSB resistance phenotype was 48.6%, 20.6%, and 35%, respectively (11, 22, 26). It is well-known that MRSA isolates are often multidrug-resistant (27). Accordingly, in this study, resistance to clindamycin and erythromycin was significantly higher in MRSA isolates than in MSSA isolates. However, the frequency of the iMLSB phenotype was significantly higher in MSSA isolates than in MRSA isolates. While more attention is paid to the treatment of infections due to MRSA isolates, MSSA should also be considered seriously. The prevalence of various *erm* genes among erythromycin-resistant *S. aureus* in Ardabil was as follows: 60.3% (in 35 isolates), 60.3% (in 35 isolates), 72.4% (in 42 isolates), and 51.7% (in 30 isolates) for *ermA*, *ermB*, *ermC*, and* ermTR* genes, respectively. The *msrA* gene was found in 9 (15.5%) erythromycin-resistant *S. aureus *isolates. In our study, the prevalence of various *erm* genes in erythromycin-resistant *S.* *aureus* was higher compared to the reported rates in Greece (28) and Belgium (29); however, it was lower than the prevalence rates reported in other studies conducted in Iran (30), as well as 2 published studies from Turkey (11, 31). In this study, several resistance gene profiles were observed. The isolates with multiple resistance-encoding genes showed higher MIC values for erythromycin. Usually, bacteria collect multiple arrays of resistance determinants to limit the activity of an antibiotic (32). 

Toxins are among the main factors contributing to the pathogenesis of *S. aureus* infections (33). Hemolysin-encoding genes (*hla *and* hld*) were the most common toxin-encoding genes identified. This result is consistent with other studies that have reported a high prevalence of hemolysin-encoding genes in *S. aureus* isolates. 

In SE-encoding genes, *sea* is the most prevalent one reported to date (33). It has been reported that SEA is responsible for most common human staphylococcal food poisoning diseases worldwide (34). Similarly, in the current study, *sea* was reported as the dominant gene encoding staphylococcal enterotoxins, while *seb, sed,* and sec genes were less common. However, *seb* encodes an enterotoxin (SEB) that is responsible for severe staphylococcal diseases beyond food poisoning (35).

TSST-1 is a superantigen responsible for a life-threatening disease, toxic shock syndrome caused by *S. aureus*. In the current study, TSST-1–encoding gene, *tst*, was detected in 33% of the isolates. Previously, the *tst* gene was reported between 1.5%-39% in clinical *S. aureus* isolates (36, 37). However, a higher frequency (78%) of the *tst *gene was reported in *S. aureus* isolates collected from healthy children (29).

In the current study, *luk* (a PVL-encoding gene) was identified in 38 (33.2%) *S. aureus* isolates. Inconsistent results have been reported in the prevalence of the *luk* gene. For instance, in a study conducted by Horváth *et al.* in Hungary, 2.3% of *S. aureus* isolates carried the *luk* gene (38), while in another study performed by Shukla *et al.* in the USA, almost all isolates were positive for the *luk* gene (39). 

The majority of isolates in the current study contained multiple toxin-encoding genes (≥3). The toxins were not found to be exclusive to MRSA or MSSA isolates. However, the frequency of some toxin genes was different between MRSA and MSSA isolates. In comparison, the rate of *hla* and *hld* genes was significantly higher in MRSA isolates, while the rate of *sed* and *eta* genes was significantly higher in MSSA isolates. This indicates that there is no absolute superiority in virulence between MRSA and MSSA isolates. Other variables may cause increased mortality in MRSA infections (38).

Spa typing is a fast and accurate method to distinguish *S. aureus *isolates in outbreaks (40). In the present study, out of the 58 erythromycin-resistant *S. aureus* isolates, 10 different spa types were identified. However, 23 (39.6) and 14 (24.1) isolates belonged to t030 and t078 spa types, respectively. This indicates partially clonal dissemination of isolates in the study settings. According to previous reports, *spa* types t030, t037, and t002 were the most common types in Asian countries, and t037 is the dominant type in Iran (41)*.* In this study, t037, the most common circulating spa type in Iran, was not detected. In a similar study performed in the same setting on *S. aureus *isolates collected from the teenage healthy student population and wastewater resources, different results were observed, in which t11332 (14.3%) and t346 (25%) were the most common spa types (29, 42).

## Conclusion

This study found a high frequency of MLSB-resistant *S. aureus* isolates with different genetic backgrounds of resistance and virulence in Ardabil hospitals. Therefore, it is important to perform D-tests routinely to detect iMLSB resistance and prevent treatment failures. Moreover, effective strategies to prevent the spread of these isolates are needed. One possible strategy could be to use antibiotics more judiciously and to implement infection control measures, such as hand hygiene and isolation precautions. Further studies are needed for better understanding the clonal relationship of the isolates to establish effective infection control measures.

## Funding

This study was financially supported by Ardabil University of Medical Sciences, Iran.

## Conflict of Interest

The authors declared no conflict of interest.

## References

[1] DeLeo FR, Chambers HF (2009). Reemergence of antibiotic-resistant Staphylococcus aureus in the genomics era. J Clin Invest..

[2] Turlej AG, Hryniewicz WA, Empel J (2011). Staphylococcal cassette chromosome mec (Sccmec) classification and typing methods: an overview. Pol J Microbiol..

[3] Maina EK, Kiiyukia C, Wamaea CN, Waiyaki PG, Kariuki S (2013). Characterization of methicillin-resistant Staphylococcus aureus from skin and soft tissue infections in patients in Nairobi, Kenya. Int J Infect Dis..

[4] Yilmaz G, Aydin K, Iskender S, Caylan R, Koksal I (2007). Detection and prevalence of inducible clindamycin resistance in staphylococci. J Med Microbiol..

[5] Delialioglu N, Aslan G, Ozturk C, Baki V, Sen S, Emekdas G (2005). Inducible clindamycin resistance in Staphylococci isolated from clinical samples. Jpn J Infect Dis..

[6] Adeyemo O, Okunye O, Nwaokorie F, Kamet O (2023). Isolation and Characterization of Coagulase Positive, Methicillin and Multi-Drug Resistant Staphylococcus and Mammaliicoccus species Isolated from Wound of Patients Attending Federal Medical Centre, Yola, Adamawa State, Nigeria. Iran J Med Microbiol..

[7] Mohanasoundaram KM (2011). The prevalence of Inducible clindamycin resistance among gram positive cocci from various clinical specimens. J Clin Diagn Res..

[8] Shrestha B, Pokhrel BM, Mohapatra TM (2009). Phenotypic characterization of nosocomial isolates of Staphylococcus aureus with reference to MRSA. J Infect Dev Ctries..

[9] Eady EA, Ross JI, Tipper JL, Walters CE, Cove JH, Noble WC (1993). Distribution of genes encoding erythromycin ribosomal methylases and an erythromycin efflux pump in epidemiologically distinct groups of staphylococci. J Antimicrob Chemother..

[10] Mittal V, Kishore S, Siddique M (2013). Prevalence of inducible clindamycin resistance among clinical isolates of Staphylococcus aureus detected by phenotypic method: A preliminary report. J Infect Dis Immun..

[11] Aktas Z, Aridogan A, Kayacan CB, Aydin D (2007). Resistance to macrolide, lincosamide and streptogramin antibiotics in staphylococci isolated in Istanbul, Turkey. J Microbiol..

[12] Schwalbe R, Steele-Moore L, Goodwin AC (2007 ). Antimicrobial susceptibility testing protocols (1st ed).

[13] CLSI (2023). Methods for dilution antimicrobial susceptibility tests for bacteria that grow aerobically. Approved standard-tenth edition, CLSI document M07-A10.

[14] Arabestani MR, Rastiyani S, Alikhani MY, Mousavi SF (2018). The relationship between prevalence of antibiotics resistance and virulence factors genes of MRSA and MSSA strains isolated from clinical samples, West Iran. Oman Med J..

[15] Abdollahi S, Ramazanzadeh R, Delami Khiabani Z, Kalantar E, Menbari S (10). Molecular detection of inducible clindamycin resistance among Staphylococcal strains isolated from hospital patients. J Ardabil Uni Med Sci..

[16] Sedaghat H, Esfahani BN, Mobasherizadeh S, Jazi AS, Halaji A, Sadeghi P (2017). Phenotypic and genotypic characterization of macrolide resistance among Staphylococcus aureus isolates in Isfahan. Iran. Iran J Microbiol..

[17] Patel M, Waites KB, Moser SA, Cloud GA, Hoesley CJ (2006). Prevalence of inducible clindamycin resistance among community- and hospital- associated Staphylococcus aureus isolates. J Clin Microbiol..

[18] Zelazny AM, Ferraro MJ, Glennen A, Hindler JF, Mann LM, Munro S (2005). et al. Selection of strains for quality assessment of the disk induction method for detection of inducible clindamycin resistance in staphylococci: a CLSI collaborative study. J Clin Microbiol..

[19] Harmsen D, Claus H, Witte W, Rothgänger J, Claus H, Turnwald D (2003). Typing of methicillin-resistant Staphylococcus aureus in a university hospital setting by using novel software for spa repeat determination and database management. J Clin Microbiol..

[20] Malachowa N, DeLeo FR (2010). Mobile genetic elements of Staphylococcus aureus. Cell Mol Life Sci..

[21] Fiebelkorn KR, Crawford SA, Mc Elmeel ML, Jorgensen JH (2003). Practical disk diffusion method for detection of inducible clindamycin resistance in Staphylococcus aureus and coagulase-negative staphylococci. J Clin Microbiol..

[22] Fokas S, Tsironi M, Kalkani M, Dionysopouloy M (2005). Prevalence of inducible clindamycin resistance in macrolide-resistant Staphylococcus. Clin Microbiol Infect..

[23] Firouzi F, Akhtari J, Nasrolahei M (10). Prevalence of MRSA and VRSA Strains of Staphylococcus aureus in healthcare staff and inpatients. J Mazandaran Univ Med Sci..

[24] Aslanimehr M, Yaghobfar R, Peymani A (2014). Detection of MLSB phenotypes and inducible clindamycin resistance in staphylococcus aureus isolates in-patients of Qazvin and Tehran teaching hospitals. J Qazvin Univ Med Sci..

[25] Fathali Z, Mirzaee M, Najarpeerayeh S (2016). Identification sec, hla, pvl and tsst-1 toxins genes profile in of Methicillin-Resistant Staphylococcus aureus clinical isolates. J Ilam Univ Med Sci..

[26] Turng B, Sinha J, Deal M, Pollitt J, Callihan D, Brasso B ( 2005). Detection and interpretation of Macrolide-Lincosamide-Streptogramin resistance among Staphylococcus with Phoenix Automated Microbiology System and BDXpert™ System. As presented at the 15th European Congress of Clinical Microbiology and Infectious Disease (ECCMID).

[27] Watkins RR, Holubar M, David MZ (2019). Antimicrobial resistance in methicillin-resistant Staphylococcus aureus to newer antimicrobial agents. Antimicrob Agents Chemother.

[28] Spilopoulou I, Petinaki E, Papandreou P, Dimitracopoulos G (2004). erm(C) is the predominant genetic determinant for the expression of resistance to macrolides among methicillin-resistance Staphylococcus aureus clinical isolates in Greece. J Antimicrob Chemother..

[29] Omid MR, Jamali H, Kafilzadeh F, Borjian A, Arzanlou M (2021). Molecular epidemiology, virulence factors, antibiotic resistance and risk factors for nasal carriage of Staphylococcus aureus in a teenage student population: High prevalence of oxacillin susceptible MRSA isolates. Jundishapur J Microbio.

[30] Hosseini SS, Niakan M, Saderi H, Motallebi M, Taherikalani M, Asadollahi K (2016). Frequency of genes encoding erythromycin ribosomal methylases among Staphylococcus aureus clinical isolates with different D-phenotypes in Tehran, Iran. Iran J Microbiol..

[31] Gul HC, Kilic A, Guclu AU, Bedir O, Orhon M, Basustaoglu AC (2008). Macrolide-lincosamide-streptogramin B resistant phenotypes and genotypes for methicillin-resistant Staphylococcus aureus in Turkey, from 2003 to 2006. Pol J Microbiol..

[32] ‏Arzanlou M, Chai WC, Venter H (2017). Intrinsic, adaptive and acquired antimicrobial resistance in Gram-negative bacteria. Essays Biochem..

[33] Otto M (2014). Staphylococcus aureus toxins. Curr Opin Microbiol..

[34] Evenson ML, Hinds MW, Bernstein RS, Bergdoll MS (1988). Estimation of human dose of staphylococcal enterotoxin A from a large outbreak of staphylococcal food poisoning involving chocolate milk. Int J Food Microbiol..

[35] Fries BC, Varshney AK (2013). Bacterial toxins-Staphylococcal enterotoxin B. Microbiol Spectr..

[36] Pinchuk IV, Beswick EJ, Reyes VE (2010). Staphylococcal enterotoxins. Toxins..

[37] Krüll M, Dold C, Hippenstiel S, Rosseau S, Lohmeyer J, Suttorp N (1996). Escherichia coli hemolysin and Staphylococcus aureas alpha-toxin potently induce neutrophil adhesion to cultured human endothelial cells. J Immunol..

[38] Horváth A, Dobay O, Sahin-Tóth J, Juhász E, Pongrácz J, Iván M (2020). Characterisation of antibiotic resistance, virulence, clonality and mortality in MRSA and MSSA bloodstream infections at a tertiary-level hospital in Hungary: a 6-year retrospective study. Ann Clin Microbiol Antimicrob..

[39] Shukla SK, Karow ME, Brady JM, Stemper ME, Kislow J, Moore N (2010). Virulence genes and genotypic associations in nasal carriage, community-associated methicillin-susceptible and methicillin-resistant USA400 Staphylococcus aureus isolates. J Clin Microbiol..

[40] Shopsin B, Gomez M, Montgomery SO, Smith DH, Waddington M, Dodge DE (1999). Evaluation of protein A gene polymorphic region DNA sequencing for typing of Staphylococcus aureus strains. J Clin Microbiol..

[41] Asadollahi P, Farahani NN, Mirzaii M, Khoramrooz SS, Van Belkum A, Asadollahi K (2018). Distribution of the most prevalent spa types among clinical isolates of Methicillin-Resistant and-Susceptible Staphylococcus aureus around the world: A review. Front Microbiol..

[42] Ranjbar Omida M, Jamali H, Kafilzadeh F, Borjian A, Arzanlou M (2023). Occurrence of Staphylococcus spp in the wastewaters from Iran: Diversity, antimicrobial resistance, and virulence potential. J Water Health..

